# Interaction between serum levels of Anti-Mullerian Hormone and the degree of sperm DNA fragmentation measured by sperm chromatin structure assay can be a predictor for the outcome of standard *in vitro* fertilization

**DOI:** 10.1371/journal.pone.0220909

**Published:** 2019-08-08

**Authors:** Peter Zarén, Sara Alson, Emir Henic, Mona Bungum, Aleksander Giwercman

**Affiliations:** 1 Dept. of Translational Medicine, Lund University, Malmö, Sweden; 2 Dept. of Obstetrics and Gynaecology, Skane University Hospital, Malmö, Sweden; 3 Reproductive Medicine Centre, Skane University Hospital, Malmö, Sweden; Universite Clermont Auvergne, FRANCE

## Abstract

Serum levels of Anti-Mullerian Hormone (AMH) have been shown to be biomarker for prediction of the quantitative aspects of ovarian reserve. On the male side, sperm chromatin structure assay (SCSA) DNA fragmentation index (DFI) has been demonstrated to be an important predictor of outcomes in standard IVF procedures but to less degree in intracytoplasmic sperm injection procedures (ICSI). The purpose of this study was to investigate whether the combination of female AMH serum levels and sperm DFI adds to prediction of the outcome of assisted reproduction. A total of 352 couples was included (ICSI-148: IVF-204) A venous blood sample was drawn for AMH analysis before IVF/ICSI treatment. DFI was measured in the ejaculate used for assisted reproduction. Regression models for the following odds ratio calculations were constructed: for obtaining at least one Good Quality Embryo; for live birth in all procedures; for pregnancy in procedures where embryo transfer was performed; for miscarriage. For DFI increase by 10 percentage points (not increased DFI as reference) odds ratio for Good Quality Embryo was statistically significantly lower when AMH was at lower quartile (AMH <12 pmol/L; OR = 0.29, 95% CI: 0.14–0.59,) but not when AMH was at upper quartile (AMH ≥ 36 pmol/L; OR = 0.95, 95% CI: 0.43–2.13,). The marginal effect of an increase in DFI by 10 percentage points was statistically significant only when AMH < 25.2 pmol/L. Similar results were obtained as considers live birth following standard IVF. No interactions were seen for standard IVF in relation to the risk of miscarriage and for any of the outcomes when ICSI was used as method of treatment. We conclude that the impact of high DFI on the outcome of standard IVF is most pronounced if the female partner has relatively low AMH levels. This finding may help in defining the role of sperm DNA integrity testing in management of infertile couples. It may also explain some of the heterogeneity in results of studies focusing on predictive value of DFI measurements in assisted reproduction.

## Introduction

In vitro fertilization (IVF) is a clinically established practice for couples experiencing infertility, annually contributing to over 100 000 infants born in Europe and 50 000 in the United States [1, 2]. However, success rates have remained low during recent years, on average, with less than a third of cycles resulting in live birth. Given the cost and the emotional distress of the procedure itself [3], a better understanding of factors predicting IVF outcomes is warranted.

Female serum levels of Anti-Mullerian Hormone (AMH) have been shown to be the biomarker of choice for prediction of the quantitative aspects of ovarian reserve, being strongly correlated to the number of antral follicles and oocyte yield in response to ovarian stimulation [4–7]. Despite this, its ability to predict outcomes in IVF procedures has so far been limited, with recent meta-analyses showing only weak associations between AMH levels and pregnancy and live birth rates, respectively [8, 9].

Factors related to male reproductive function are assumed to play a role in as many as 50% of infertility cases [10]. From a clinical point of view, it is, therefore, plausible to take both markers of female and male fertility into account when predicting the outcome of applying assisted reproduction techniques (ART).

Sperm chromatin structure assay (SCSA) DNA fragmentation index (DFI) expressed as %DFI, i.e., the percentage of sperms in the sample that have increased red fluorescence that corresponds to DNA strand breaks, has recently been demonstrated to be an important predictor of IVF outcomes in standard IVF procedures but not in intracytoplasmic sperm injection procedures (ICSI) [11]. Ovarian reserve tests such as serum AMH levels have been shown to be of limited value in predicting IVF outcomes in the presence of unexplained or male infertility [12], prompting the need to take male factors into account when predicting IVF outcomes using AMH. Even though attempts to develop such models have previously been made [13, 14], their definition of “male factor” has been based on the evaluation of sperm concentration, motility and morphology, parameters known not to be powerful predictors of *in vitro* fertility [15]. The purpose of this study was therefore to investigate whether the combination of female AMH serum levels and sperm DFI adds to improved prediction of the outcome of assisted reproduction.

## Materials and methods

### Study design and patient population

A retrospective cohort study was performed, analyzing both ICSI and standard IVF outcomes in relation to female AMH serum levels and sperm DNA fragmentation index (DFI) measured at treatment start. The cohort was based on a previous database, consisting of 456 couples undergoing IVF procedures at the Reproductive Medicine Centre (RMC), Skåne University Hospital, Malmö, Sweden, between 2010 and 2016, for which AMH serum levels were measured at start of treatment. For each couple, only their first attempted cycle at the clinic was considered.

**Fig 1:**
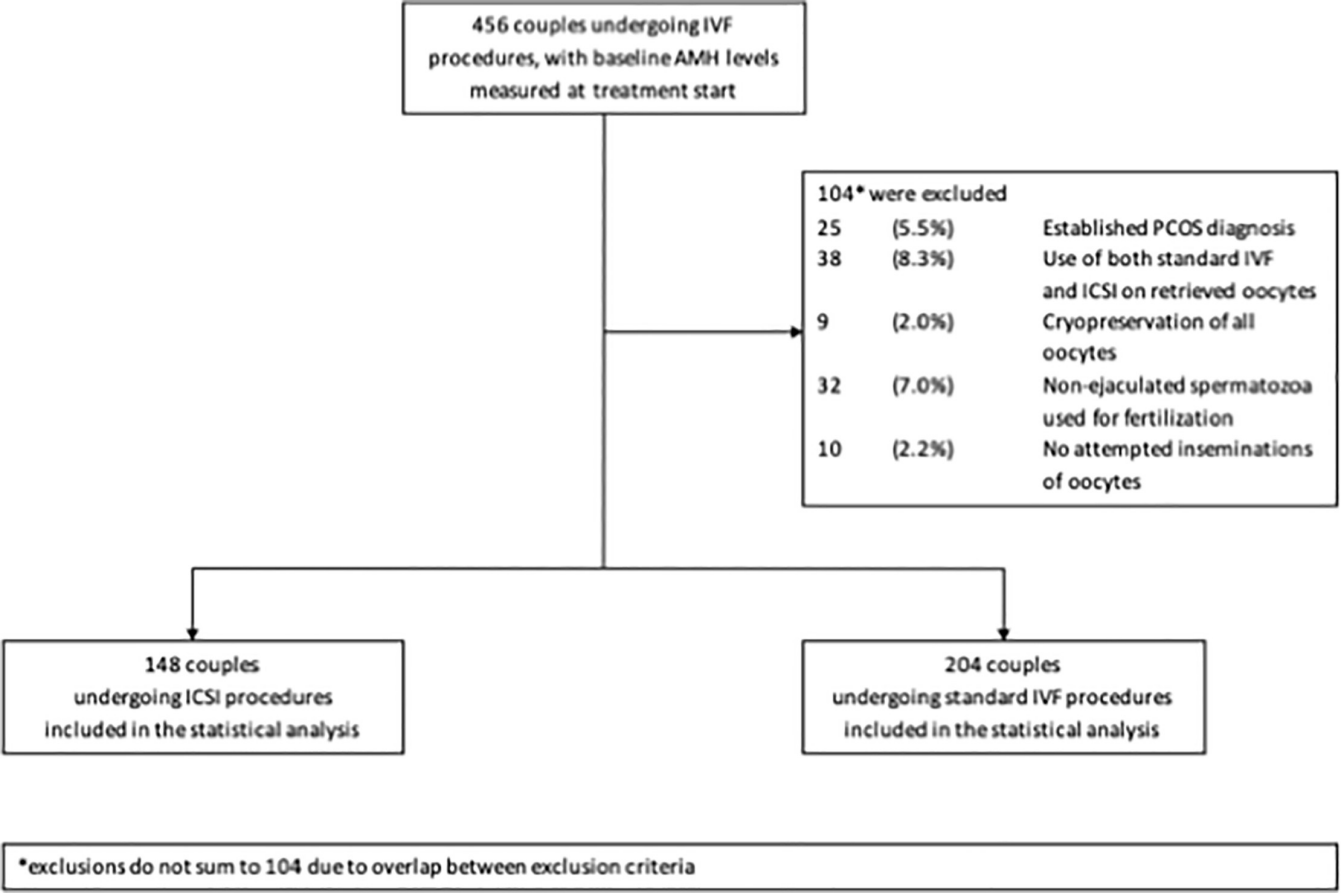
Flowchart for inclusion/exclusion of study participants.

A total of 352 couples was included in the study, with 148 couples undergoing ICSI procedures and 204 couples undergoing standard IVF procedures. Exclusion criteria included an established PCOS diagnosis (N = 25), the use of both standard IVF and ICSI during the same cycle (N = 38), cryopreservation of all oocytes (N = 9), the use of non-ejaculated spermatozoa for fertilization (N = 32) and the absence of attempted inseminations of oocytes (N = 10). In total– 104 couples fulfilled one or more of the exclusion criteria. Inclusions and exclusions of participants are depicted in Fig 1.

General eligibility criteria for IVF procedures at the Reproductive Medicine Centre included female age < 40 at start of treatment, female body mass index (BMI) < 30 as well as both partners being non-smokers. Demographics of the ICSI and standard IVF groups, as well as the group of excluded couples, are shown in Table 1. The sperm concentration varied between the excluded group (median 27.5 ×10⁶/mL), the IVF group (median 55.5 ×10⁶/mL) and the ICSI group (median 13.0 ×10⁶/mL), but the groups did not differ in terms of body mass index (BMI), male or female age. The levels of serum AMH for different female age quartiles are shown in Table 2. The AMH levels of the different age groups varied, with median AMH ranging from 16 pmol/L (ages 37–39) to 27 pmol/L (ages 22–30).

**Table 1:** The characteristics of study participants and excluded couples.

	Participants IVF n = 204	Participants ICSI n = 148	Excluded couples n = 104
Female age (years), median, /range	33.5/25-39	32/22-39	32/20-39
Female BMI (kg/m^2^) median/range	22.8/16.7–30.0	23.6/18.4–29.8	23.1/18.8–30.9
Male age (years), median, /range	35/24-48	33/21-54	34/24-53
Sperm concentration (x10^6^/mL), median/range	55.5/5.6–270.0	13.0/0.1–203.0	27.5/0.2–170.0

**Table 2:** Serum AMH levels according to female age quartiles.

	22–30 years	31–33 years	34–36 years	37–39 years
AMH (pmol/L) median/range	27/0-137	18.5/0-117	20/0-91	16/0-95

The study was approved by the Ethical Committee of Lund University and all couples have given an informed written consent.

### AMH measurement procedure

A venous blood sample was drawn for AMH analysis before IVF/ICSI treatment. Serum was isolated and stored at -80° C, until analyzed at the Department of Clinical Chemistry, Skåne University Hospital in Malmö, Sweden. Serum levels of AMH were measured using the Electro Chemi Luminiscence Immuno assay (ECLI) provided by Roche Elecsys AMH. The lowest detectable level was 0.07 pmol/L, and coefficients of variation were 2% at 6.86 pmol/L.

### Semen collection and analysis

Sperm samples were collected by masturbation 1 hour before oocyte retrieval. Semen analysis were performed within 1 hour from the time of ejaculation according to WHO guidelines [16]. One hundred μL of the raw semen sample was frozen at –80°C for SCSA analysis.

### Sperm chromatin structure assay

SCSA analysis was done as previously described [17–19]. All measurements were performed on raw semen, which on the day of analysis was quickly thawed and analyzed immediately. Initially, a 30 s treatment with a pH 1.2 buffer denatures the DNA at the sites of single- or double-strand breaks, whereas normal double-stranded DNA remains intact. Subsequently, the fluorescent DNA dye—Acridine orange—is added. After blue light excitation in a flow cytometer, denaturated (single-stranded) DNA emits red fluorescence and the intact (double-stranded) DNA emits green fluorescence. Sperm chromatin damage is quantified by the flow cytometry measurements of the metachromatic shift from green (native, double-stranded DNA) to red (denatured, single-stranded DNA) fluorescence and displayed as red versus green fluorescence intensity cytogram patterns. The extent of DNA denaturation is expressed as the DFI (%DFI), which is the percentage of sperm in the sample that have increased red fluorescence that corresponds to DNA strand breaks. The frequency histogram of DFI provides a more precise calculation of percentage DFI than the use of computer gating on the green versus red cytogram.

Using FACSort (Becton Dickinson, San Jose, CA, USA) five thousand spermatozoa per ejaculate were analyzed. Thereafter, we processed the flow cytometric data using SCSASoft (SCSA Diagnostics, Brookings, SD, USA). The same reference sample–run for every fifth measurement—was used for the instrument setup and calibration during the whole study period. The intra-laboratory CV for DFI analysis was found to be 4.5%.

### IVF and ICSI procedures

Ovarian stimulation was performed according to either an antagonist or agonist protocol. Stimulation protocol was chosen according to individual patient characteristics. For ovarian hyperstimulation, recombinant FSH, either Follitropin alfa, (GONAL-f, Merck-Serono, Darmstadt, Germany), Follitropin beta, (Puregon, Organon, Ireland Ltd), Urofollitropin, (Fostimon, Institut Biochimique SA (IBSA), Lugano, Switzerland), Chorifollitropin alfa, (Elonva, Merck Sharp & Dome, (MSD), New Jersey, USA), or human menopausal gonadotropins (hMG) (Menotropin, Menopur, Ferring, GmbH, Kiel, Germany) in individual doses were used. Follicle development was monitored by vaginal ultrasound. When three or more follicles reached 17 mm, ovulation was induced with human chorionic gonadotropin (hCG) (Ovitrelle, Merck, KGsA, Darmstadt, Germany), and transvaginal follicle aspiration was performed 35–36 hours later.

### Assessment of fertilization, embryo morphology classification, cryopreservation and embryo transfer

Aspirated oocytes were assessed, and either inseminated or injected with sperm depending on semen quality parameters. Fertilization was recorded 18 hours after IVF/ICSI. Embryos were assessed and defined as Good Quality Embryos (GQE) on day 2 or 3 [20] or on day 5 according to the Gardner blastocyst grading scale [21].

Embryo transfer (ET) was performed two, three or five days after ovum pick up (OPU).

### Luteal phase support, pregnancy test and miscarriage

Luteal phase support with vaginal gel with micronized progesterone (Crinone 8%, Merck) was given for two weeks after OPU. Commercial urine hCG pregnancy test kit was used 12–14 days after embryo transfer to assess conception, and pregnancy was confirmed with vaginal ultrasound in gestational week > 7. Miscarriages and births were registered. Pregnancy loss before week 22 was considered a miscarriage. Live birth was defined as delivery of one living child in gestational week > 22.

### Statistical analysis

All statistical analyses were performed using R version 3.4.0 [22] with the addition of packages ‘Hmisc’ and ‘rms’ [23, 24]. All calculations were done separately for ICSI and standard IVF. Odds ratios (OR) were calculated using binary logistic regression models with DFI and baseline AMH as predictors, allowing for interaction between DFI and AMH by also including a product term. To handle cases with missing data (N = 76, 21.6%), multiple imputation was performed using the ‘aregImpute’ algorithm in the ‘rms’ package. For the imputation process, all model predictors and outcome variables were used, as well as the parameters female BMI, type of ART used (IVF/ICSI), sperm motility and maternal and paternal ages.

Regression models for the following OR calculations were constructed:

1. OR for obtaining at least one good quality embryo (GQE) during a procedure.
2. OR for live birth in all procedures where injection/insemination of oocytes was attempted.
3. OR for pregnancy in procedures where embryo transfer (ET) was performed. For this calculation, 49 procedures in which no embryos of good quality were obtained were excluded, using the remaining 303 procedures for the analysis.
4. OR for miscarriage in procedures where pregnancy was achieved. For this analysis, only the 157 procedures in which pregnancy was achieved were included.

All regression models were assessed for interaction between AMH and DFI by performing Wald tests for additivity. For outcomes where interaction was statistically significant, a second regression model was constructed where also the number of aspirated oocytes as well as a product term DFI × aspirated oocytes were included as predictors.

The marginal effects of DFI at varying levels of AMH are presented as the OR of each outcome for DFI increased by 10 (the interquartile range), percentage points, e.g. from 20% to 30%, vs not increased (reference). AMH quartiles used for these interquartile range change (IQR change) measures were calculated from the whole cohort of included couples (n = 352). P-values < 0.05 were used for statistical significance.

## Results

### Obtaining at least one good quality embryo

**Fig 2:**
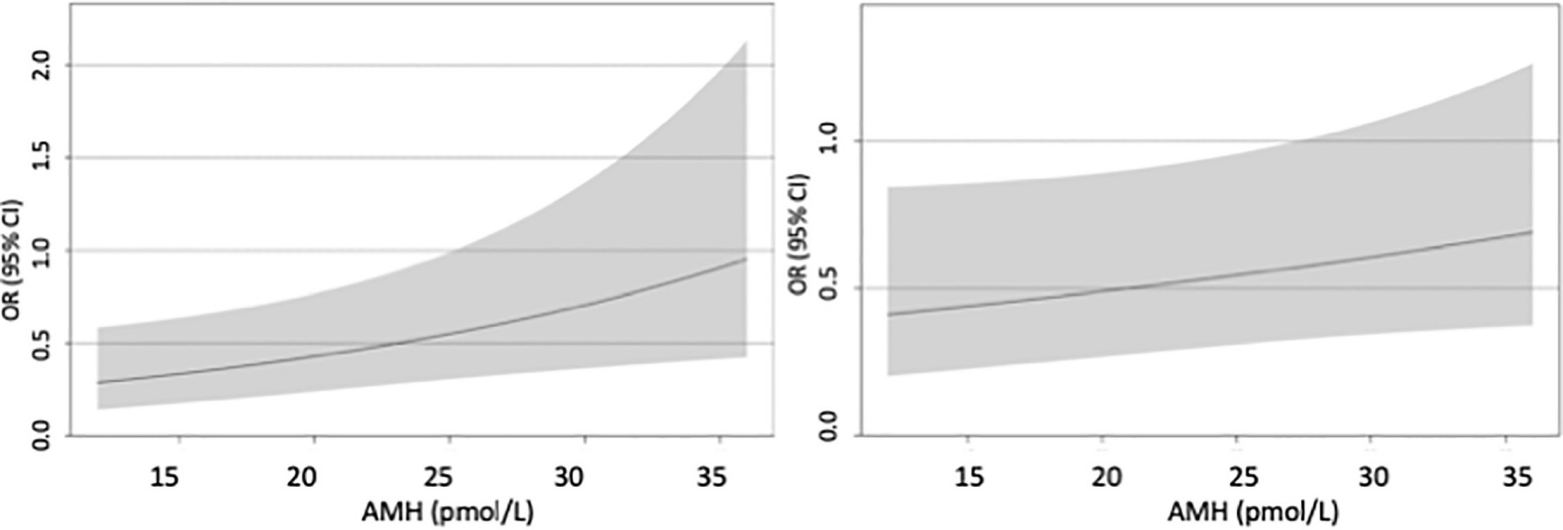
Odds ratio for obtaining at least one good quality embryo during standard IVF procedures for DFI increased by 10 percentage points vs not increased (reference). Displayed for interquartile range values of AMH. Grey area indicates 95% confidence intervals. a) not adjusted for oocyte yield; b) adjusted for oocyte yield displayed for interquartile range values of AMH when oocyte yield is 9 (median).

Interaction between AMH and DFI was statistically significant for the outcome of obtaining at least one good quality embryo when using IVF (p = 0.017), where also non-interaction terms of AMH and DFI had a statistically significant impact on the outcome (p = 0.036 and p = 0.001, respectively). This interaction is illustrated in Fig 2A, where the effect of DFI on the outcome is presented at varying levels of AMH. OR for at least one GQE when DFI increased by 10 percentage points (the IQR change) vs not increased (reference) is lower when AMH is low (OR = 0.29, 95% CI: 0.14–0.59, at lower quartile AMH <12 pmol/L) than when AMH is high (OR = 0.95, 95% CI: 0.43–2.13, at upper quartile AMH ≥ 36 pmol/L). The marginal effect of an increase in DFI by 10 percentage points was statistically significant only when AMH < 25.2 pmol/L.

Interaction between AMH and DFI was statistically significant also for the oocyte yield-adjusted analysis for obtaining at least one good quality embryo when using IVF (p = 0.023), where also non-interaction terms of AMH and DFI had a statistically significant impact on the outcome (p = 0.022 and p = 0.016, respectively). The oocyte yield-adjusted interaction between AMH and DFI is illustrated in Fig 2B, where the effect of DFI on the outcome is presented at varying levels of AMH when oocyte yield is 9 (median). OR for GQE when DFI increased by 10 percentage points (the IQR change) vs not increased (reference) was statistically significantly lower when AMH was at lower quartile (AMH < 12 pmol/L; OR = 0.30, 95% CI: 0.14–0.62,) but not when AMH was at upper quartile (AMH ≥ 36 pmol/L; OR = 0.91, 95% CI: 0.40–2.04,).

No interaction between AMH and DFI for the outcome of obtaining at least one GQE was observed when using ICSI.

### Live birth

**Fig 3:**
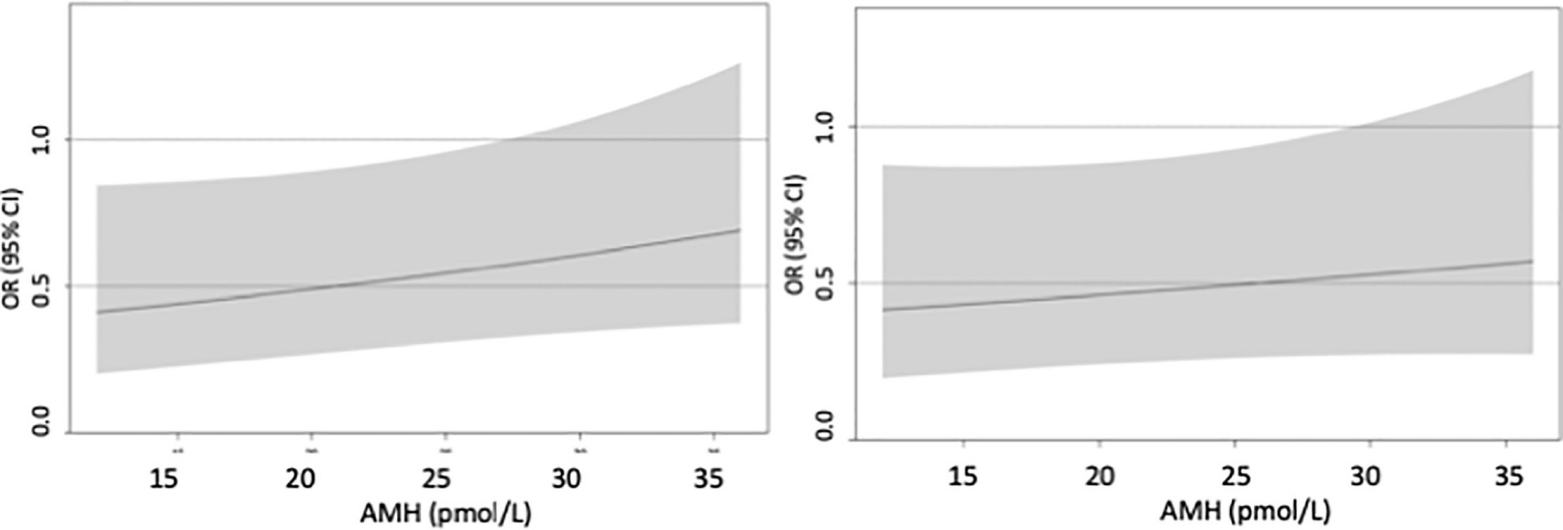
Odds ratio for live birth in standard IVF procedures where insemination of oocytes was attempted for DFI increased by 10 percentage points vs not increased (reference). Displayed for interquartile range values of AMH. Grey area indicates 95% confidence intervals. a) not adjusted for oocyte yield; b) adjusted for oocyte yield displayed for interquartile range values of AMH when oocyte yield is 9 (median).

Statistical significance was not achieved for the interaction between AMH and DFI when using IVF (p = 0.14). However, as the acquisition of at least one GQE (where interaction between AMH and DFI was observed for IVF) is an intermediary step towards achieving live birth, the effect of DFI on achieving live birth using IVF is still presented at varying levels of AMH in Fig 3A. The OR for live birth for DFI increased by 10 percentage points (the IQR change) vs not increased (reference) was lower when AMH was low (OR = 0.41, 95% CI: 0.20–0.84, at lower quartile AMH <12 pmol/L) than when AMH was high (OR = 0.69, 95% CI: 0.38–1.27, at upper quartile AMH ≥ 36 pmol/L). The marginal effect of an increase in DFI by 10 percentage points was statistically significant only when AMH < 27.3 pmol/L.

Similarly, despite not achieving statistical significance for interaction in the non-oocyte-yield-adjusted analysis, the oocyte yield-adjusted effect of DFI on achieving live birth using IVF is presented at varying levels of AMH when oocyte yield is 9 (median) in Fig 3B. In this analysis, statistical significance was not achieved for interaction between AMH and DFI (p = 0.42). The oocyte yield-adjusted OR for live birth for DFI increased by 10 percentage points (the IQR change) vs not increased (reference) was statistically significantly lower when AMH was at lower quartile (AMH < 12 pmol/L; OR = 0.42, 95% CI: 0.20–0.88) but not when AMH was at upper quartile (AMH ≥ 36 pmol/L; OR = 0.57, 95% CI: 0.28–1.18,).

No interaction between AMH and DFI was observed when using ICSI.

### Pregnancy and risk of miscarriage

No interaction between AMH and DFI was observed for the outcomes of obtaining pregnancy after embryo transfer or the risk of miscarriage given pregnancy, neither for IVF nor ICSI.

## Discussion

Our study demonstrated that serum AMH levels modified the association between sperm DFI, as assessed by means of SCSA, and the chance of obtaining at least one GQE and that of live birth, when applying standard IVF. This was, however, not case for ICSI treatments.

Previous studies have indicated that high DFI may have a negative impact on the outcome of natural [25, 26] and assisted reproduction [27]. Furthermore, some reports–including our own data–found this effect to be more pronounced when using IVF and not ICSI as the method of fertilization [11, 28]. Our current data fit with these findings, but the novel aspect is showing that at high AMH levels the chance of positive outcome of IVF treatments seems not to be affected by high DFI.

Analyzing interaction between AMH and DFI, statistically significant interaction was only found when one or more GQE but not live birth was used as an outcome. However, our post hoc analysis of the live birth outcome showed a congruent although weaker trend, with high levels of AMH reducing the negative impact of high DFI. This suggests the lack of statistical significance for the interaction (p = 0.14) when live birth was used as an outcome could be due to low statistical power.

Interestingly, the analyses were robust for adjusting for the number of aspirated oocytes. Thus, it might indicate that our findings cannot simply be explained by a correlation between AMH levels and the number of fertilized oocytes, instead suggesting a connection between AMH levels and oocyte quality.

The observed interaction between AMH levels and DFI was found for outcomes in which the analysis included all couples where insemination/injection of the oocyte(s) was attempted.

In contrast, no interaction could be found when considering pregnancy rates and the risk of miscarriage, outcomes where success in obtaining a good quality embryo for transfer was a prerequisite for inclusion in the analysis.

This indicates that the impact of the interaction between DFI and AMH on IVF outcome is related to early stages of fertilization. Low AMH levels might, therefore, reflect lower capacity of the oocyte to repair the numerous sperm DNA strand breaks implied by high DFI, resulting in a disturbed fertilization process following standard IVF. High female age might be one of the determinants leading to low AMH as well as decreased reparative capacity of the gametes [29, 30]. In our cohort, older women tended to have decreased levels of AMH, as expected, compared to younger women.

Our findings might also, at least partly, explain the less pronounced impact of high DFI on the ICSI outcome as compared to that of IVF. Normal AMH levels are more frequently found among females undergoing ICSI as compared to those treated with standard IVF [31], this difference possibly being due to the fact that majority of ICSI treatments are offered to couples with decreased male fertility.

Our study has some strengths and weaknesses. This is, to our knowledge, the first study showing that the impact of DFI on success rate in *in vitro* assisted reproduction, is dependent on the levels of an oocyte-related marker. This information may be of significant value in developing future predictive models for outcome of IVF treatments. Due to the relatively limited power of the study, some of the negative findings might be due to a type 2 error.

In our analysis we decided not to adjust for the age and BMI of the female. The reason is that both of them have an impact on the levels of AMH and including them in the statistical model would imply an over-adjustment. The design of the study excluded women with an established PCOS diagnosis. Therefore, the way AMH levels modulate the association between sperm DFI and standard *in vitro* fertilization success–found in our study–should be understood in this context. DFI measurement as performed with SCSA show moderate correlation with outcome of other sperm DNA integrity tests as e.g. COMET and TUNEL. Thus, it remains to be elucidated whether our findings can be extrapolated to other sperm DNA fragmentation tests.

In conclusion, we found that the impact of high DFI on the outcome of standard IVF is most pronounced if the female partner has relatively low AMH levels. This finding may help in defining the role of sperm DNA integrity testing in management of infertile couples. It may also explain some of the heterogeneity in results of studies focusing on predictive value of DFI measurements in assisted reproduction [32].
